# Co-designing primary and secondary outcome measures for an international phase 3 trial of surgical de-escalation in vulvar cancer through a consumer workshop

**DOI:** 10.1186/s40900-026-00902-8

**Published:** 2026-06-15

**Authors:** Lauren Angel, Monika Janda, Andrew Martin, Brianna Armstrong, Merran Williams, Nicola Du Thaler, Emma Wild, Mary Li, Andreas Obermair

**Affiliations:** 1 Faculty of Health, Medicine and Behavioural Sciences, The University of Queensland, Brisbane, QLD Australia; 2 Brisbane, QLD Australia; 3 Royal Brisbane and Women’s Hospital, Queensland Centre of Gynaecological Research, Brisbane, QLD Australia

**Keywords:** Patient engagement, Lived experience, Shared decision-making, Gynaecological oncology, Lymphoedema, Treatment burden, Quality of life

## Abstract

**Background:**

Consumer involvement in clinical trials has developed to be a critical driver of feasibility and real-world translation. In gynaecological oncology, patient and clinician priorities can differ, particularly regarding acceptable risks and treatment burden. Vulvar cancer treatment typically involves a groin lymph node dissection, which carries significant morbidity, effecting the long-term health of patients. To address these harms, the Australian National Vulvar Cancer (ANVU) Phase 3 international randomised clinical trial (NCT06476639) will compare standard upfront groin lymph node dissection with serial groin ultrasound monitoring, a less invasive option, to reduce treatment burden without compromising survival. In this paper, research methodologists collaborated to bring consumer perspectives into the trial design to ensure the outcomes reflect what matters most to patients.

**Main body:**

We conducted a structured co-design workshop involving patients with lived experience of vulvar cancer as well as a multidisciplinary research team. The workshop explored priorities across three stages of care to determine the outcome measures for a clinical trial: before treatment, during treatment, and after treatment. Priority in the short-term was complete cancer removal, with acceptance of some short-term adverse effects. Medium-term priorities focused more on quality of life and functional recovery, as well as fear of recurrence. While long-term priorities identified avoiding chronic harms such as lymphoedema and maintaining a normal daily life, as important. These insights informed the trial’s primary endpoint (survival following nodal metastasis at 30 months) and patient-centred secondary outcomes such as health-related quality of life, return to usual activities, and reduced chronic harms.

**Conclusions:**

Patient priorities shift as they progress through the various stages of cancer treatment, highlighting the need for dynamic, patient-centred clinical trial designs. The ANVU trial aims to incorporate true co-design in complex surgical oncology research. With consumers as research partners, the team has produced a trial protocol that is scientifically robust as well as grounded in lived experience, increasing its potential for real-world impact. The current paper focuses on co-design of the outcome measures of the trial, however consumer involvement will remain ongoing throughout the ANVU trial to ensure that the research outcomes are accessible and enable women to be provided with treatment options which suit their needs.

## Abstract (plain language summary)

Clinical trials have traditionally focused of patient survival and been driven by researchers to identify areas of need. Medical research is shifting toward a patient-centred approach, prioritising what matters most to those directly affected. This paper outlines how researchers and women with lived experience of vulvar cancer, a rare cancer affecting the skin and tissue of the vulvar, collaborated to design the outcome measures for an international clinical trial. The trial aims to reduce treatment burden without increasing cancer risk, to ensure women can maintain quality of life after cancer. The collaborative workshop explored what mattered most at three stages of care: before treatment, during treatment, and after treatment. Revealing patient priorities shift over time, with complete cancer removal being a priority in early treatment, functional recovery and fear of recurrence as medium-term priorities, and wellbeing considered in the longer-term. By embedding these insights into the clinical trial, the research team aim to influence everyday clinical decisions and support women to make treatment choices that best suit their individual needs and improve wellbeing.

## Background

Consumer involvement has shifted from a research afterthought to a recognised driver of relevance, feasibility and translation in clinical trials [1]. When consumers shape research questions and outcome measures, trials are more likely to target real-world problems, recruit successfully and report findings that matter to patients rather than just clinicians [2, 3]. Early consumer involvement has been shown to reduce avoidable trial amendments, improve acceptability of study procedures and sharpen ethical decision-making by highlighting burdens that clinical teams often underestimate [4].

Consumer involvement can occur through activities such as targeted consultation, advisory roles or full co-design [5]. While full co-design may not always be feasible, structured engagement with people who have lived experience reliably uncovers priorities that would otherwise be missed [6]. In gynaecological oncology, consumers have drawn attention to unmet informational needs, treatment burdens and the consequences of disease and treatment on daily life [7, 8]. Patients and clinicians do not always share a similar view of acceptable risks. Among 117 vulvar cancer patients, 66% preferred a complete inguino-femoral lymphadenectomy in preference to a 5% false-negative rate of the sentinel node procedure. In contrast, 60 out of 100 gynaecologists were willing to accept a 5–20% false-negative rate of the sentinel lymph node procedure [9]. Our own consumer-guided qualitative research demonstrated that women with vulvar cancer consistently consider avoidance of lymphoedema, clear communication, shared decision-making and minimising treatment trauma against survival outcomes and prognosis [10]. These insights highlight the limitations of conventional trial designs that focus solely on survival while overlooking what patients identify as life-defining.

Vulvar cancer is uncommon and standard care for stage IB–II disease involves surgical excision of the primary tumour and sentinel node biopsy (SNB) where eligible, or inguinofemoral lymphadenectomy (IFLND) when SNB is not possible [11]. Although SNB reduces morbidity, only around half of patients meet eligibility criteria [12, 13]. Both SNB and IFLND are associated with substantial short- and long-term harms, including wound complications, infection and chronic lymphoedema [14–16]. These consequences shaped the strong consumer preferences identified in our previous work, where fear of lymphoedema outweighed fear of the cancer itself for some patients [10].

To address these harms, we propose to replace upfront groin lymph node dissection (LND) for Stage IB and II vulvar cancer patients with serial high-resolution ultrasound monitoring (USM) to detect groin metastases. A phase 3, international, randomised clinical trial will be conducted (https://www.clinicaltrials.gov/ | NCT06476639 | 2024/06/17), where eligible patients with a negative baseline groin ultrasound will be randomised to receive either upfront groin LND (either SNB or IFLND as per local practice management guidelines, standard treatment arm) or serial groin USM for up to 18 months (intervention treatment). In the intervention group, groin LND will only be required for the few women with ultrasound-detected positive nodes when they are still small. This strategy aims to spare selected patients the morbidity of groin surgery while maintaining an acceptably low risk of mortality from nodal metastasis. Whether this trade-off is safe and acceptable is the central question of our trial.

During the development of the trial protocol, the investigator group consulted with consumers about which primary and secondary treatment outcomes genuinely reflect what matters most to patients. This prompted the present co-design workshop, designed to directly involve both consumers and methodologists in determining trial outcome measures. Building on our earlier qualitative findings [10], the workshop aimed to identify which clinical and patient-centred outcome measures should anchor the trial, ensuring that the design reflects both methodological robustness and the lived priorities of people affected by vulvar cancer.

This manuscript describes the methods and outputs of that workshop as a key step in embedding consumer perspectives into a major international clinical trial, with the aim of improving both scientific validity and real-world impact.

## Methods

In September 2025, the clinical trial research team met with six health consumers with lived experience of vulvar cancer, a rare gynaecological cancer. The research team comprised two gynaecological oncologists, an epidemiologist, two biostatisticians, a biomarker scientist and two clinical trial staff. Researchers were included in the workshop to help ensure that the endpoints which were selected were statistically valid and achievable given the available resources.

The health consumer group reflected a range of lived experience. Four women had been diagnosed with cancer between 2018 and 2022, and were 4–8 years post-treatment at the time of the workshop (diagnosed between ages 50–64 years old). These consumers did not have a SNB prior to treatment, with one having had a needle aspiration biopsy with no nodal involvement detected. Two consumers had a bilateral ILFND during initial treatment. One consumer was the husband and primary support person of a woman treated for vulvar cancer, offering a valued carer perspective. The sixth consumer was an experienced consumer research partner and former oncology Registered Nurse living with the long-term effects of ovarian cancer treatment. Her insights, combined with more than a decade of coordinating a gynaecological cancer support group, contributed important depth and external consumer perspectives to the discussion. Three of the consumers in the workshop had developed lymphoedema due to their treatment. Consumers were invited based on their treatment experiences (Table 1) and their previous involvement in, or expression of interest in contributing to, vulvar cancer research.

**Table 1:**
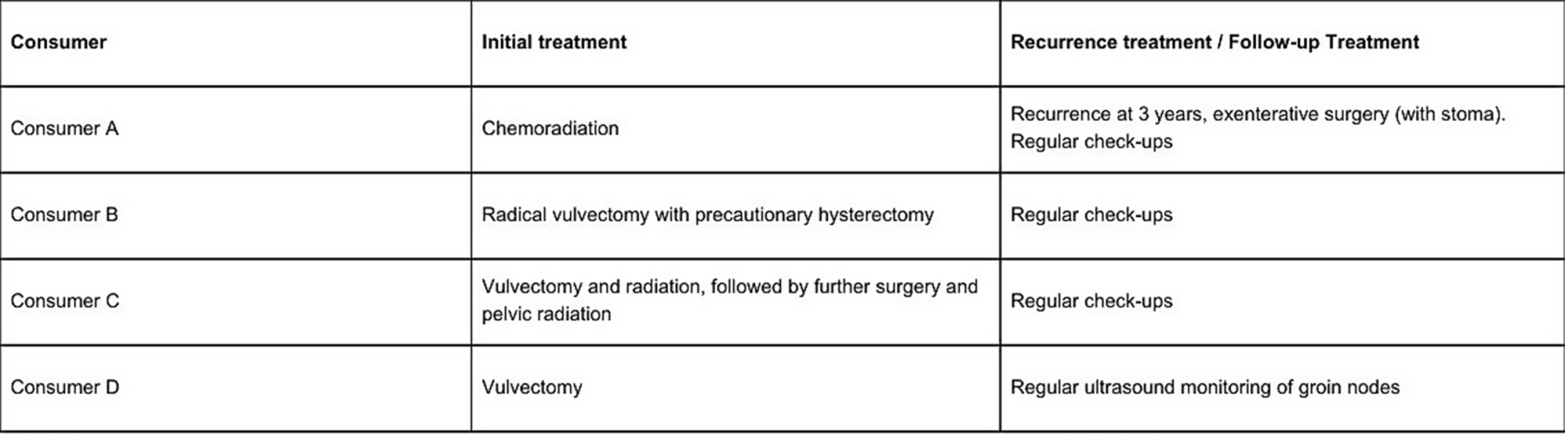
Treatment experience for vulvar cancer health consumers involved participating in the workshop

The workshop discussion was co-facilitated by a behavioural and implementation scientist with expertise in conducting qualitative methods and a long-standing track record in consumer engagement, alongside the experienced consumer research partner who has contributed to multiple research projects across universities and institutions in Queensland, Australia. Together, the facilitators helped foster a supportive environment, guide group dynamics and enable consumers felt comfortable to contribute openly and meaningfully.

The structure for the workshop, along with previous and ongoing consumer engagement activities, was guided by the Metro North Health co-design framework which includes the stages: Engage and Align; Explore and Connect; Imagine and Decide; Create and Test; Co-implement and Co-evaluate; and Share and Celebrate [5]. This approach helped ensure that we involve the right people at the right times and prepare them effectively to contribute meaningfully at each stage.

**Fig. 1:**
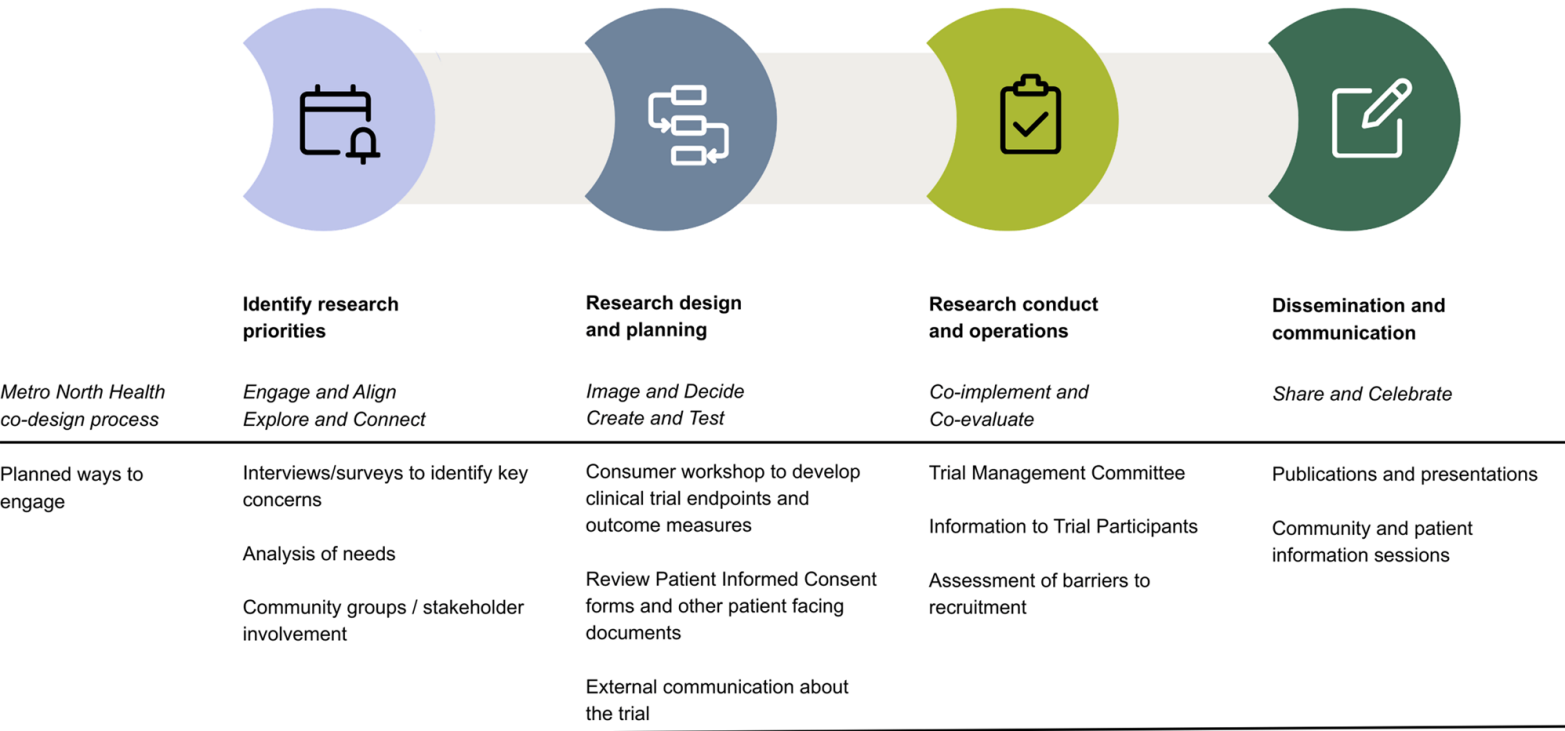
Overview of the consumer engagement strategy underpinning the ANVU clinical trial, structured according to the Metro North Health co‑design framework

Our broader consumer strategy for the ANVU clinical trial aims to embed consumer involvement throughout the entire research process. Consumers contribute during the design phase through interviews and focus groups (Create and Test), during implementation through their roles on the Trial Management Committee (Co-implement and Co-evaluate), and during dissemination through co-authorship and participation in community and patient-facing sessions (Share and Celebrate). This strategy is illustrated in Fig. 1.

Prior to the present workshop, a Consumer-Led Research Group, comprised of four women with lived experience of vulvar cancer and two carers (Engage and Align) undertook a thematic analysis of interviews held with 10 women treated for vulvar cancer [10]. This early work helped identify key concerns and priorities related to diagnosis, treatment and survivorship. These findings, together with input from the Lymphoedema Association Australia and Cherish Women’s Cancer Foundation, informed the development of the initial research question (Explore and Connect).

The purpose of the present workshop was to co-design the primary and secondary outcome measures for the clinical trial. These outcome measures were not predetermined. Rather, the earlier qualitative work highlighted important themes that were brought to the workshop discussion, including the need to capture meaningful long-term outcomes.

Pre-reading materials were provided to consumers seven days in advance of the workshop to allow time to review the information and prepare any clarifying questions for the team. These materials included an overview of what clinical trials are and why they are conducted, along with general examples of potential trial endpoints and outcome measures (kept intentionally non-specific to avoid influencing the consumers). We also explained why consumer involvement is critical to the success of this project. Some aspects of the trial design, including the research question, aim, study population, and intervention, had already been developed in collaboration with our Consumer-Led Research Group and Vulvar/Vaginal Cancer Consumer Group. This information was summarised in the pre-reading materials. All materials were written in plain, consumer-friendly language, avoiding unnecessary scientific terminology, to ensure consumers had a clear understanding of the purpose of the clinical trial and the specific role of the workshop, as one aspect of the trial’s design.

Prior to the workshop, the research team received guidance outlining the expectations of the workshop and suggested approaches for behaviour to ensure that the consumers felt comfortable to voice their opinions openly and confidently. We recognise that an inherent power differential exists between the health consumers and researchers, and we sought to minimise its impact by fostering an environment where all workshop participants were encouraged to contribute equally and work collaboratively in the development of solutions and a shared goal. For example, researchers were introduced based on their expertise and contribution rather than their role or position, and only plain language, or terminology already familiar to consumers through the pre-reading materials, was used during the workshop. Informal follow-up conversations with consumers after the workshop confirmed that they felt comfortable contributing, that their views were heard, and that their perspectives were meaningfully incorporated into the workshop outcomes.

The workshop began with all attendees introducing themselves and providing an overview of their professional expertise or lived experience in relation to the topic. This was followed by an experienced consumer research partner who spoke about the importance of consumer involvement in clinical trials. The consumers participating in the workshop reflected a range of research experience, from individuals engaging for the first time to others who had been contributing to research activities since their treatment.

To facilitate discussion, attendees were then divided into three small groups to explore three scenarios. Group 1 comprised five health consumers, Group 2 included members of the research team, and Group 3 consisted of the experienced health consumer and a researcher who were participating online. This structure enabled initial discussion on each topic among the consumers without researcher input. Whole-workshop discussions were moderated by the two co-facilitators, supporting consumers to speak about their perspectives.

First, the consumers were invited to reflect on the time of their diagnosis (pre-treatment), including treatment options presented to them by their doctor/s, and what short- and long-term outcomes were most important for them when considering treatment options prior to making those initial decisions. Researchers were asked to contribute to the discussion by considering a hypothetical scenario where a loved one has been diagnosed with cancer and to reflect on which outcomes they would view as most important to them.

Secondly, members of the workshop were asked to consider whether their priorities changed during their treatment.

Thirdly, workshop participants were asked if they would rank their treatment outcomes differently at a time when they completed their cancer treatment; and knowing what they now know about treatment impacts and outcomes, i.e. the short- and long-term impacts of treatment.

## Results

**Fig. 2:**
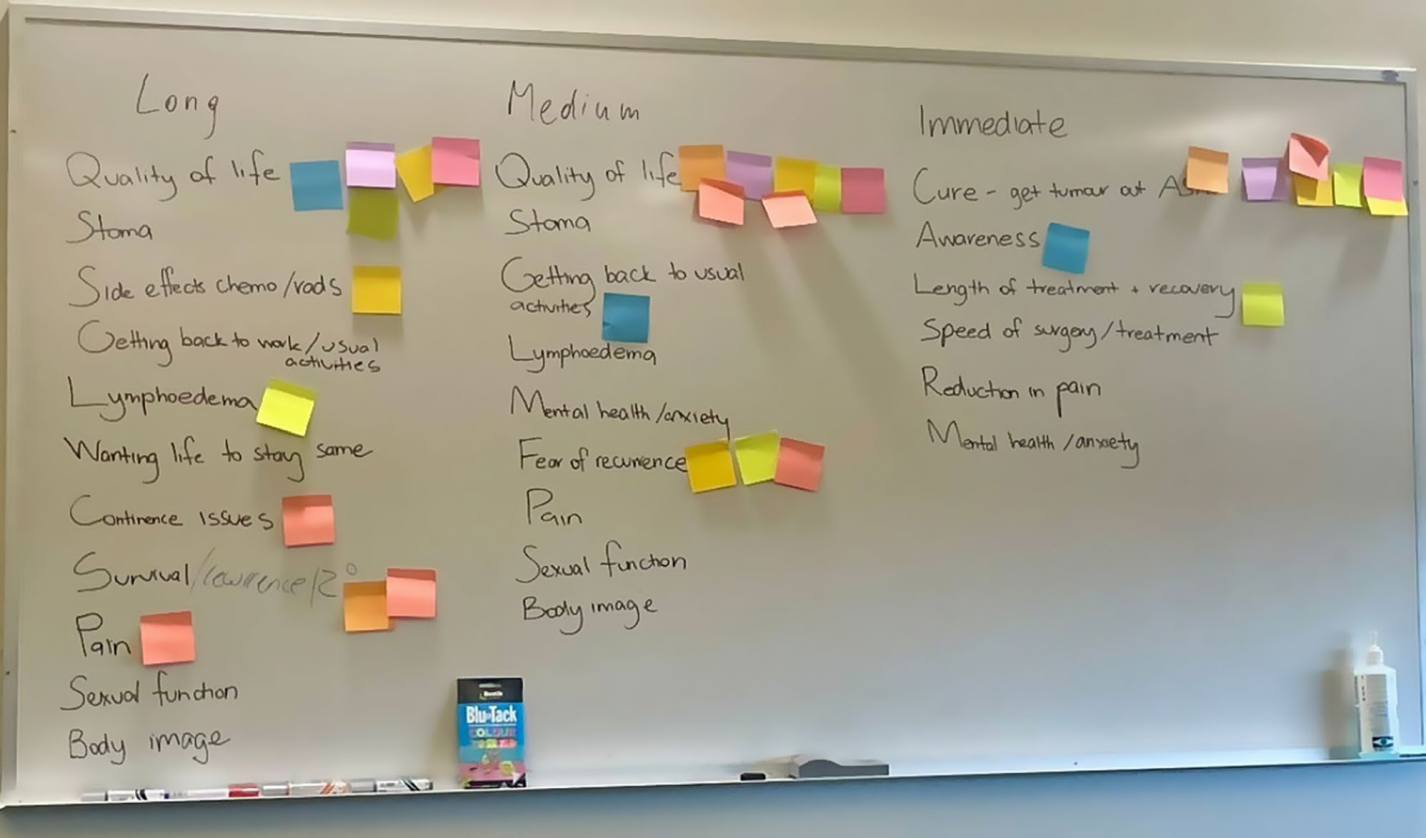
Quantified consumer priorities across the short-, medium-, and long-term stages following vulvar cancer diagnosis. Each sticky-note represents one vote from a workshop participant. Colours indicate vote only and are not associated with any other meaning

The outcomes identified during the workshop discussions were summarised and categorised as short-, medium- and long-term to determine their relevance at each stage of cancer diagnosis, treatment, and recovery. All attendees were then provided with three sticky-notes (colours were not associated with individuals) and invited to select a single outcome in each category that they considered to be the highest priority (Fig. 2). Voting was conducted openly, allowing the workshop participants to see the collective preferences as they emerged.

In the short-term and with regards to the perioperative phase, consumers felt that removing the primary vulvar cancer surgically in its entirety, as soon as possible, was giving them the greatest chance of long-term cure. In the perioperative setting, dealing with pain, recovery, and length of hospital stay were less important. In brief, consumers were prepared to accept short-term adverse effects to maximise long-term survival.

In the medium-term, referring to the months following surgery (up to 12 months), quality of life and the ability to perform usual daily activities dominated consumers preferences. Fear of recurrence was an additional concern that affected consumers’ quality of life during the medium-term.

In the long-term (12 + months from surgery) priorities were more varied. Being able to lead a normal life without long-term side effects from surgery (such as lymphoedema) or chemotherapy or radiation treatment, maintaining urinary continence, and being pain free were the most mentioned priorities. When they were disease-free during this time, consumers were hopeful they could remain disease-free in the long-term and lead a “normal” life.

Based on the outcomes prioritised during the voting process, the workshop attendees developed the primary and secondary endpoints for the ANVU clinical trial. Consistent with phase 3 clinical trials, focusing on effectiveness as primary outcome measure, survival secondary to the development of groin node metastasis at 30 months was chosen as the primary endpoint for the ANVU trial. This endpoint was selected because the development of groin node metastases in vulvar cancer occurs early (up to 18 months post-surgical treatment), and recurrence is associated with a high mortality (90%) despite subsequent treatment [17]. A 30-month endpoint allows sufficient time for clinically meaningful differences to emerge. The secondary outcome measures, identified by the consumers as important to patients, include various dimensions of health-related quality of life, ability to resume usual daily activities, lower limb lymphoedema, pain, morbidity, and fear of recurrence. Together, these outcomes reflect both clinical relevance and the lived experiences emphasised by our consumers.

Following the workshop, all attendees were provided with a written summary of the meeting notes to review, ensuring that their contributions were accurately documented. The consumers were also encouraged to provide feedback on their experience participating in the co-design process, further reinforcing their perspectives were valued by the research team. While voting was not anonymous, the post-workshop follow-up allowed the consumers an opportunity to reflect on their experience and express objections or changes to their selected endpoints anonymously.

## Discussion

This workshop highlighted that the priorities of women with vulvar cancer shifts markedly across the trajectory of care. Early in treatment, consumers prioritised one thing above all else: complete cancer removal, even if it meant a difficult perioperative journey. As treatment progressed, their focus shifted toward maintaining quality of life, the ability to get back to their normal routines, and the ever-present worry that the cancer might return. In the long-term, avoiding lymphoedema and other chronic treatment-related harms was central to their idea of a life worth living.

These evolving priorities directly shaped the final outcomes set for the ANVU phase 3 trial. Survival following nodal metastasis was established as the primary endpoint, consistent with the expectations for a definitive effectiveness trial. The secondary outcomes reflect the realities that patients have described [10] and were raised as concerns by the consumers in the workshop. Measures of quality of life, functional recovery, lower limb lymphoedema, pain, morbidity, and fear of recurrence are no longer optional extras. They are core indicators of whether the intervention delivers value where it matters.

While the researchers had personal insights from prior gynaecological cancer trials, no outcomes were pre-specified by the wider research team. The final objectives and endpoints were driven by the health consumers at the workshop. Importantly, the selected outcomes align with the scientific and regulatory expectations for a phase 3 clinical trial, and are achievable to measure with the available resources.

As vulvar cancer is a rare disease, we were only able to identify a small number of consumers who were interested in engaging in structured, reflective discussions about trial design and outcome prioritisation. Consequently, only a small number of consumers were available to contribute to this stage of the trial design process. We acknowledge the workshop consumers represent a narrow demographic range, with all attendees based in Queensland, Australia, and that this group cannot capture the full demographic, geographic or clinical diversity of individuals affected by vulvar cancer. To partially address this limitation, the workshop included an experienced consumer who has facilitated a gynaecological cancer support group for over 15 years, as well as a partner/carer participant. While this does not substitute for broader representation, it provided additional contextual insight and helped situate individual experiences within a wider spectrum of lived experience.

Consumer input was based on retrospective experience, with the most recent diagnosis among the group occurring four years prior. While recall or reconstruction bias is a recognised limitation, we sought to mitigate this by structuring the discussion around specific stages of the cancer journey (diagnosis, treatment, and follow-up) rather than relying on general recollection [18–20]. The time elapsed since diagnosis did enable our consumers to reflect on preferable long-term outcomes that only become fully apparent with distance from treatment.

## Conclusion

The workshop demonstrated that meaningful co-design is possible in complex surgical oncology research when consumers are well-prepared, respected and engaged as genuine partners. Their contributions enhanced our trial design and unsettled a few assumptions. For example, while the researchers knew survival is critically important for consumers, we realised that it is equally important to them that their quality of survival is high. Ultimately this process has produced a trial that is more grounded in lived experience, more relevant to what matters to patients, and more likely to translate beyond academic papers into everyday clinical decision-making.

Ongoing consumer involvement will remain an essential component of the ANVU trial in line with our consumer engagement strategy. Co-design is not a single event but a continuing commitment to working alongside those who live with the consequences of cancer and its treatment. If clinical trials are to meaningfully inform practice, instead of results sitting on a shelf, this collaborative approach is the direction we need to keep taking.

## Data Availability

Not applicable.

## Abbreviations

IFLND: Inguinofemoral lymphadenectomy
LND: Lymph node dissection
SNB: Sentinel node biopsy
USM: Ultrasound monitoring
